# Changes in the mean echogenicity and area of the puborectalis muscle during pregnancy and postpartum

**DOI:** 10.1007/s00192-015-2905-4

**Published:** 2015-12-16

**Authors:** Anique T. M. Grob, Mariella I. J. Withagen, Maria K. van de Waarsenburg, Karlijn J. Schweitzer, Carl H. van der Vaart

**Affiliations:** Department of Reproductive Medicine and Gynecology, University Medical Center, PO box 85500, 3508 GA Utrecht, Netherlands; MIRA Institute for Biomedical Technology and Technical Medicine, University of Twente, Enschede, Netherlands

**Keywords:** 3D/4D ultrasound, Area, Echogenicity, Pelvic floor

## Abstract

**Introduction and hypothesis:**

Three-dimensional (3D) and four-dimensional (4D) volume transperineal ultrasound imaging is increasingly used to assess changes in the dimensions of the pelvic floor during pregnancy and after delivery. Little is known with regard to the area of the puborectalis muscle and its structural changes. Echogenicity measurement, a parameter that provides information on the structure of muscles, is increasingly used in orthopaedics and neuromuscular disease evaluation. This study is aimed at assessing the changes in the mean echogenicity of the puborectalis muscle (MEP) and the puborectalis muscle area (PMA) during first pregnancy and after childbirth.

**Methods:**

The MEP and PMA of 254 women during first pregnancy were measured at 12 and 36 weeks’ gestation and 6 months postpartum. To determine the effect of child-birth on MEP and PMA, the results at 6 months postpartum were separately analysed for vaginal deliveries, operative vaginal deliveries (ventouse) and caesarean section deliveries. Mean differences in MEP and PMA were analysed using ANOVA statistics.

**Results:**

The MEP at 6 months postpartum was, independent of manoeuvre, significantly (*p* < 0.001) lower than MEP values during pregnancy. After caesarean delivery, the PMA was significantly smaller at maximum pelvic floor contraction than PMA after vaginal delivery (*p* = 0.003) or operative vaginal delivery (*p* = 0.002).

**Conclusion:**

Our study indicates that structural changes in the puborectalis muscle during and after pregnancy, as measured by MEP, occur and can be analysed. In addition, the mode of delivery affects the area of the puborectalis during contraction after delivery. For true volume analysis, as part of an assessment of contractility of the puborectalis muscle we will need 3D volume analysis.

## Introduction

Pregnancy and childbirth are known risk factors for the development of pelvic floor disorders such as pelvic organ prolapse (POP) and urinary incontinence [1, 2]. Ultrasound imaging, and especially three-/four-dimensional (3D/4D) transperineal ultrasound, has contributed to our understanding of the anatomical changes that are involved in the pathophysiology of these symptoms [3, 4]. At present, data collected with ultrasound are either objective measurements such as hiatal dimension, bladder neck position, and levator urethral gap distances, or subjective observations such as levator ani avulsions or muscular haematomas [5–9]. Recently, we described a method of reliably measuring the puborectalis muscle area (PMA) and mean echogenicity of the puborectalis muscle (MEP) [10]. Echogenicity is used to diagnose neuromuscular diseases in children without the need for tissue biopsy to discriminate between myopathies and neuropathies, in orthopaedics to analyse supraspinatus tendon tears, and in animal studies as an indicator of muscle healing processes [11–14].

The levator ani muscle, and especially the puborectalis part, is important in closing the genital hiatus and thereby offering support to the pelvic organs and ligaments [15]. Information about the composition of the puborectalis muscle by measuring its echogenicity during and after pregnancy may add to our understanding of the effect of pregnancy and delivery on pelvic floor function.

The aim of our study was to measure changes in the PMA and MEP using 3D/4D transperineal ultrasound in women during and after their first pregnancy.

## Materials and methods

### Study design and population

Over a period of 2 years, 280 nulliparous pregnant women were seen for 3D/4D transperineal ultrasound assessment of their pelvic floor anatomy during and after pregnancy. The current study is part of this larger prospective observational study on the association between pelvic floor symptoms and changes in pelvic floor anatomy during and after first pregnancy [16, 17]. Women were excluded when they had a medical history of urinary and/or faecal incontinence, previous prolapse or anti-incontinence surgery, connective tissue disease or neurological disorders. The Institutional Human Research Ethics Committee approved the study and all women gave informed consent.

### Ultrasound examination

The assessment consisted of 4D transperineal ultrasound imaging using a GE Voluson 730 Expert system (GE Healthcare, Zipf, Austria) with an RAB 4- to 8-MHz curved array volume transducer. The angles of the acquired volume are set 85° longitudinal and 70° transverse to the probe, a temporal resolution of 3 Hz was used to acquire the data, and settings that might influence the intensity values were kept constant for each measurement (e.g. gain 15, power 100, Harmonics mid, contrast 8, grey map 4, persistence 8, enhance 3, depth 6 cm, 1 focus point, at a fixed height according to preset, time gain compensation (TGC) in a straight line in the centre). All pelvic floor ultrasound examinations were performed with the participants in the supine position and with an empty bladder [5]. The ultrasound probe was placed on the perineum in the sagittal plane. Measurements were taken with the musculature at rest, during contraction and during Valsalva at approximately 12 weeks’ gestation, 36 weeks’ gestation and 6 months after delivery. The data sets were stored on a hard disk for offline processing.

### Image reconstruction and analysis

Offline analysis of the data was performed using 4D View 7.0 (GE Medical Systems Kretztechnik, Zipf, Austria) and Matlab® R2010a (MathWorks, Natick, MA, USA) by two of the authors (ATMG and MKW). Observers were blinded to the delivery mode during postprocessing of the data. Image analysis was carried out by first determining and fixing the point of time of the muscle at rest, at maximal muscle contraction and during Valsalva (4D data turned into 3D data). The plane of minimal hiatal dimensions is selected following the guidelines [5, 18, 19]. This plane is used to obtain tomographic ultrasound images (TUI) in the axial direction. The first slice in which the pubic bones are closed is used for analysis. This 2D ultrasound image contains 1,304 × 662 pixels and is exported as a .bmp file to Matlab® R2010a (Image Processing Toolbox 7.0).

### Delineation of structures

**Fig. 1:**
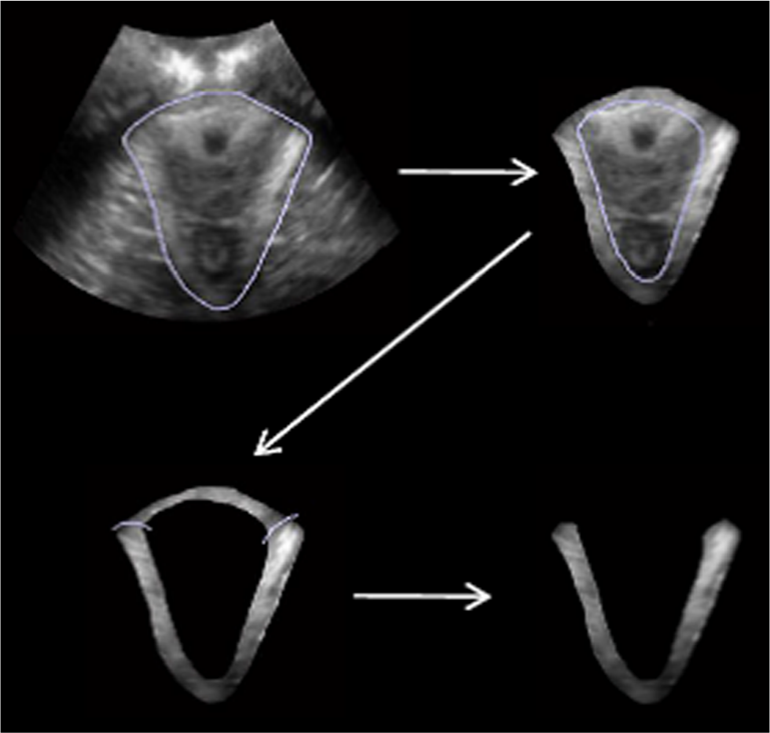
Delineation of the puborectalis muscle by hand

The region of interest (ROI), the puborectalis muscle, was delineated semi-automatically using the software Matlab (function “imfreehand”) as described previously and visualised in Fig. 1 [10]. The PMA is calculated by multiplying the number of pixels within the ROI with the size of one pixel (cm^2^).

Echogenicity is based on the grey-scale image in which the value for each pixel can vary between 0 (black) and 255 (white). Normal muscle cells are echolucent and appear dark on the image. The connective and fatty tissues around and within the muscle have a higher echogenicity and appear brighter [20]. The MEP was determined automatically by calculating the sum of the echogenicity of each pixel and dividing that number by the number of pixels. We recently demonstrated that the interobserver reliability is moderate for measuring PMA and almost perfect for the MEP [10]. For both PMA and MEP the intraobserver reliability is almost perfect.

### Statistical analysis

Data collected at 12 and 36 weeks’ gestation and 6 months postpartum were compared. The data at 6 months postpartum were separately analysed for women who delivered vaginally, women who had an operative vaginal delivery (ventouse) and those who delivered by caesarean section. The PMA and MEP of the groups were compared using ANOVA statistics. To correct for the number of comparisons made, the level of statistical significance level was adjusted using the Bonferroni method. (ANOVA *p* = 0.05/9 comparisons, Bonferroni-adjusted *p* = 0.006).

## Results

Of the 280 women recruited from the clinic, 26 were excluded. Two women were incorrectly included (1 had a twin pregnancy and the other had a neurological disorder), 1 woman had an immature delivery at 19.9 weeks’ gestation, 17 women were excluded based on loss to follow-up and/or missing ultrasound volume datasets because of technical errors during file saving (at least 2 out of 3 datasets were missing), and 6 datasets were excluded because the symphysis was located outside the view of the ultrasound images.

The mean age of the women was 31.1 years (SD: 4.1) and their mean body mass index (BMI) at 12 weeks’, 36 weeks’ gestation and 6 months postpartum was 23.4 (SD 3.9), 27.6 (SD 3.8) and 24.0 (SD 3.9) kg/m^2^ respectively. Mean gestational age at first visit (weeks) was 13.3 (SD 1.9), at second visit 36.0 (SD 0.9) weeks and 40.2 (SD 1.6) weeks at delivery.

Of the 254 women included, optimal data analysis at 12 weeks’ gestation was possible for 247 cases at rest, 240 during contraction and 223 during Valsalva. At 36 weeks’ gestation these numbers were 219, 206 and 194, and postpartum 226, 195, 186 respectively.

Of the 254 women included, 47 (18.5 %) underwent a caesarean section, 157 (61.8 %) had a spontaneous vaginal delivery, 45 (17.7 %) had a ventouse operative vaginal delivery (15 based on fetal distress, 15 based on failure to progress, 9 based on a combination of failure to progress and fetal distress and 6 with an unknown reason for ventouse extraction) and in 5 patient files the mode of delivery was not recorded.

In the caesarean group, 11 women had an elective caesarean section, 14 had an emergency caesarean section owing to foetal distress and 17 had an emergency caesarean section because of failure to progress. Additionally, in 5 women the indication for the caesarean section was fetal distress combined with failure to progress.

### MEP

**Fig. 2:**
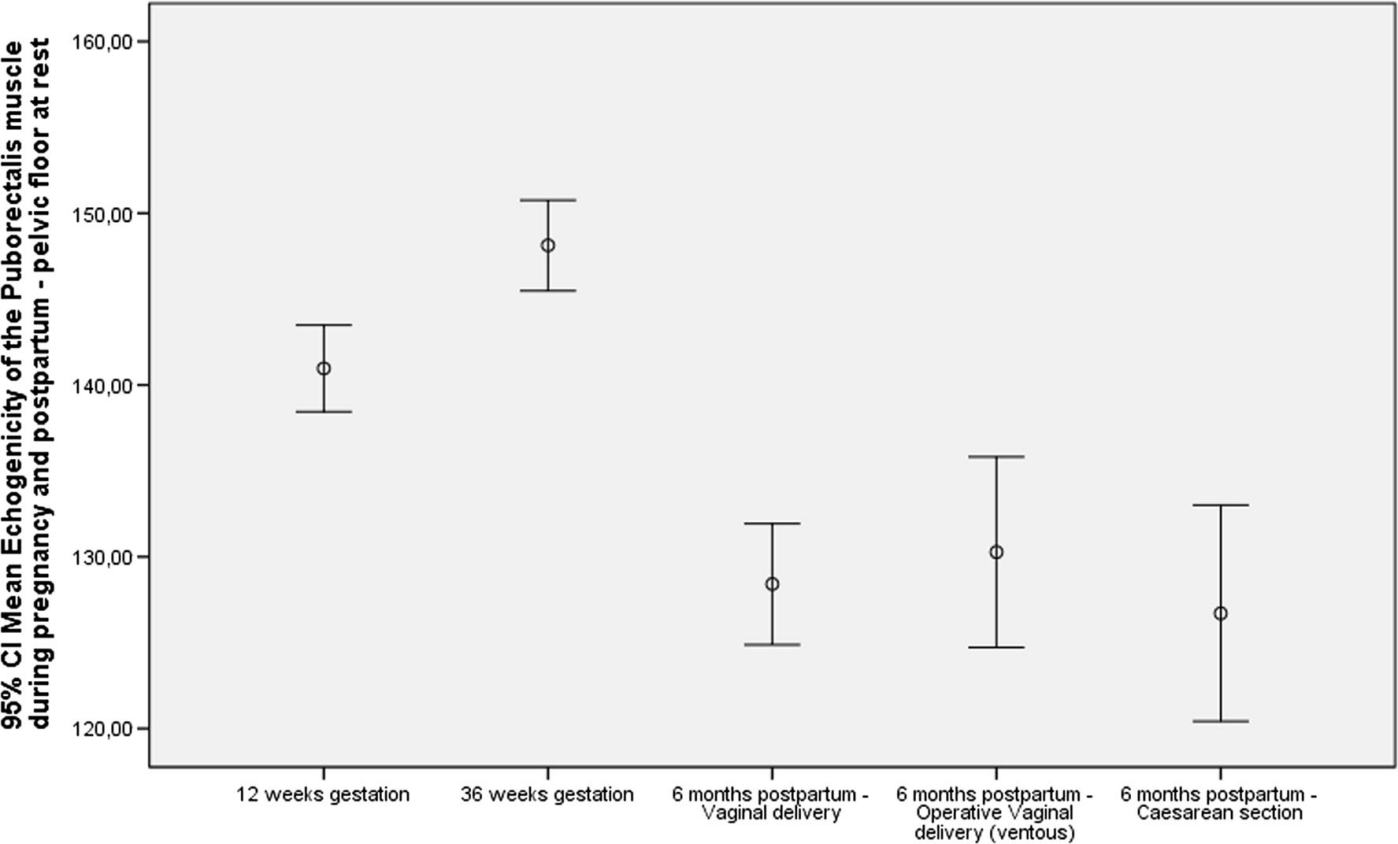
Mean echogenicity of the puborectalis muscle (MEP) during pregnancy and postpartum: pelvic floor at rest

The MEP values at 6 months postpartum were all, independent of manoeuvre, significantly lower (*p* < 0.001) than MEP values during gestation (Table 1; Fig. 2). No differences were found between the different modes of delivery. In addition, MEP values were significantly higher at 36 weeks’ gestation than at 12 weeks, with the pelvic floor muscles at rest and during contraction.

**Table 1:** Mean echogenicity of the puborectalis muscle (MEP) values during pregnancy and postpartum

MEP	At 12 weeks’ gestation	At 36 weeks’ gestation	At 6 months postpartum		
			Vaginal delivery	Ventouse delivery	Caesarean section
	*n*, mean (SD)	*n*, mean (SD)	*n*, mean (SD)	*n*, mean (SD)	*n*, mean (SD)
At rest	247, 141 (20)	219, 148, (20)	144, 128 (21)	40. 130 (17)	43, 127 (20)
During contraction	240, 133 (21)	206, 138 (21)	121, 122 (23)	33, 122 (23)	41, 116 (23)
During Valsalva	223, 135 (21)	194, 134 (23)	114, 115 (22)	31, 113 (20)	41, 123 (21)

### PMA

**Fig. 3:**
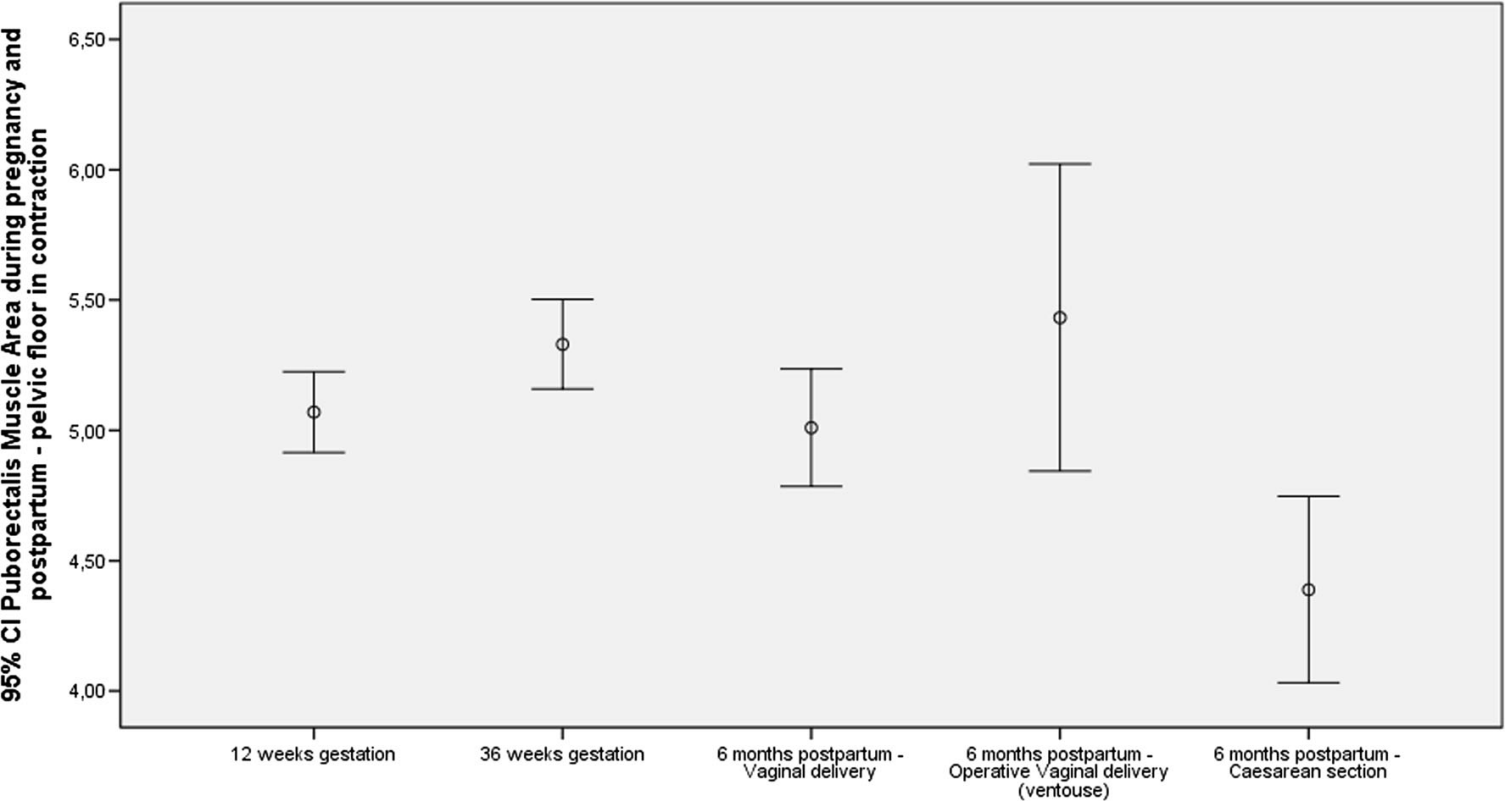
Puborectalis muscle area (PMA) during pregnancy and postpartum: pelvic floor during contraction

Tables 2 and 3 show that women at 6 months postpartum who had a caesarean section have a significantly smaller puborectalis muscle area during contraction compared with vaginal delivery (*p* = 0.003) and operative vaginal deliveries (*p* = 0.002; Fig. 3). There was no significant difference in PMA between the vaginal delivery group and women who had an operative vaginal delivery. The PMA at 6 months postpartum is significantly larger at rest (*p* = 0.004), during contraction (*p* = 0.004) and during Valsalva (*p* = 0.001) compared with PMA at 36 weeks’ gestation. Additionally, the PMA at 12 weeks’ gestation during Valsalva is significantly larger (*p* < 0.001) than PMA at 36 weeks’ gestation.

**Table 2:** Puborectalis muscle area (PMA) values during pregnancy and postpartum

PMA	At 12 weeks’ gestation	At 36 weeks’ gestation	At 6 months postpartum		
			Vaginal delivery	Ventouse delivery	Caesarean section
	*n*, mean (SD) (cm^2^)	*n*, mean (SD) (cm^2^)	*n*, mean (SD) (cm^2^)	*n*, mean (SD) (cm^2^)	*n*, mean (SD) (cm^2^)
At rest	247, 5.62 (1.35)	219, 5.85 (1.33)	144, 5.48 (1.35)	40, 5.88 (1.55)	43, 5.17 (1.20)
During contraction	240, 5.07 (1.21)	206, 5.33 (1.25)	121, 5.10 (1.35)	33, 5.43 (1.66)	41 4.38 (1.10)
During Valsalva	223, 5.84 (1.35)	194, 6.36 (1.42)	115, 5.83 (1.43)	31, 6.28 (1.63)	41, 5.71 (1.41)

**Table 3:** Mean difference in PMA and MEP values during pregnancy and postpartum; mean difference in MEP and PMA values between different types of delivery (vaginal ventouse and vaginal caesarean section)

	12–36 weeks’ gestation	36 weeks’ gestation to 6 months postpartum (general)	12 weeks’ gestation to 6 months postpartum (general)	6 months postpartum		
				Vaginal versus operative vaginal	Vaginal versus caesarean	Operative vaginal versus caesarean
	Mean difference (*p* value)	Mean difference (*p* value)	Mean difference (*p* value)	Mean difference (*p* value)	Mean difference (*p* value)	Mean difference (*p* value)
Rest						
MEP	6.88 (<0.001)	−19.6 (<0.001)	−12.7 (<0.001)	−1.86 (0.61)	2.2 (0.53)	3.55 (0.40)
PMA (cm^2^)	−0.23 (0.07)	0.37 (0.004)	0.14 (0.25)	−0.40 (0.11)	0.39 (0.09)	0.71 (0.02)
Contraction						
MEP	5.2 (0.005)	−17.1 (<0.001)	−11.9 (<0.001)	0.09 (0.98)	6.8 (0.10)	6.55 (0.22)
PMA (cm^2^)	−0.26 (0.02)	0.37 (0.004)	0.11 (0.37)	−0.32 (0.18)	0.71 (0.003)	1.04 (0.002)
Valsalva						
MEP	−0.5 (0.821)	−17.8 (<0.001)	−18.3 (<0.001)	1.56 (0.72)	−8.1 (0.03)	−9.50 (0.05)
PMA (cm^2^)	−0.52 (<0.001)	0.49 (0.001)	−0.03 (0.84)	−0.45 (0.14)	0.21 (0.39)	0.57 (0.11)

## Discussion

We studied the effect of pregnancy and delivery on the MEP and PMA using 3D/4D transperineal ultrasound. During pregnancy MEP significantly increased from 12 to 36 weeks’ gestation when measured with the pelvic floor muscle at rest and during contraction. After delivery MEP significantly decreased compared with pregnancy values. We observed a significantly smaller PMA during contraction at 6 months after caesarean delivery than in women who had a vaginal delivery.

To appreciate our findings several issues need to be discussed. The observation that there is a significantly higher MEP during pregnancy than postpartum may be explained in several ways. The first, and most suggestive, explanation is that during pregnancy MEP increases as a result of changes in the intramuscular balance between muscle cells and connective or fatty tissue, in favour of the more echogenic connective or fatty tissue. This theory is supported by the observation that as early as the first stage of gestation, when fetal growth is very limited; the body aims to store nutrients for future demands [21]. The increased pregnancy levels of progesterone act as an insulin antagonist, which causes, together with the increased intake of nutrients, an increase in intracellular and intramuscular fat storage [22]. This observation is supported by the work of Herrera, who reported accumulation of fat during pregnancy in humans and in rats [21]. As Reimers and co-workers described that fat replacement is the main cause of increased muscle echogenicity, this increased lipogenesis may well explain our findings of the significant increase in echogenicity during pregnancy and the decrease at 6 months after delivery [23]. It would have been instructive if baseline values for nulliparous non-pregnant women had been available; however, these were not obtained in this study. The second possible explanation for the changes in MEP lies in the distance and angle between the ultrasound probe and the target organ, the puborectalis muscle. When ultrasound waves travel through a deeper or different tissue composition, the returning waves have different characteristics. These differences could result in a change in echogenicity [24]. However, the effect of increasing probe pressure to the women’s perineum, shortening the distance between the probe and the puborectalis muscle, produced no significant changes in MEP when at least normal pressure to the perineum was applied [25]. The effect of the transducer angle to the muscle should also be considered. Given that the reflection of an acoustic ultrasound wave is strongest when the propagation direction of the wave is perpendicular to muscle fibres [26], this would suggest a different orientation of the ultrasound beam to the fibres of the puborectalis muscle during pregnancy. We found no supporting literature to indicate a major difference in muscle angle during pregnancy; therefore, the expected difference in angulation of the puborectalis muscle in comparison to the ultrasound beam is minor.

The final possible explanation for the difference between MEP during gestation and at 6 months after delivery is that delivery itself changes MEP. In this scenario we assume that the pre-pregnancy MEP is equal to the MEP during pregnancy, and that delivery trauma itself results in a decrease in MEP. As we did not observe a difference in MEP between women who had a vaginal delivery and those who had a caesarean delivery, we do not find this hypothesis plausible.

We found a significantly smaller PMA during contraction, but not at rest, at 6 months after caesarean delivery compared with vaginal delivery. This outcome should be interpreted within the perspective of the physical law of consolidation of mass. This law indicates that a decrease or increase in volume, in our case the puborectalis muscle, in two directions, will always affect the volume in the third dimension in the opposite way. The smaller muscle area during contraction indicates that in the plane we analysed the puborectalis muscle could be contracting better after a caesarean delivery than after a vaginal delivery. However, for true volume analysis, as part of an assessment of contractility of the puborectalis muscle we will need 3D volume analysis. The same issue of consolidation of mass holds true for explaining the larger PMA at 12 weeks’ gestation during Valsalva compared with 36 weeks’ gestation.

We did not find a significant difference in MEP or PMA between the vaginal delivery and operative vaginal delivery. This indicates that the passage of the child’s head itself causes the changes in PMA, and that this effect is not exaggerated by the use of instrumental delivery. In the literature there is some controversy about the long-term consequences of a ventouse delivery. In multiparous women the ventouse delivery was reported to be associated with pelvic floor symptoms [27]. In contrast, a study performed in primiparous women showed no additional effect of a ventouse delivery on symptoms [28]. This would be in line with our results, showing no difference in PMA and MEP between ventouse and normal vaginal delivery.

A limitation of this study was that we could not collect complete datasets for all women included. Some women withdraw from the study (because of pregnancy-related problems or non-attenders), others had an early delivery (before the second visit) or were lost to follow-up without reason. The second reason for incomplete datasets was the inability of some women to perform a maximum contraction or Valsalva. As described by Orejuela and co-workers, a maximum Valsalva should at least have a duration of >6 s [29]. We did not specify this duration in our study protocol. Together with our clinical observation that third-trimester pregnant women are less willing or are unable to perform a maximum Valsalva, we believe that our data on Valsalva have to be interpreted with caution.

One of the major strengths of our study is its prospective design, including measurements at 12 and 36 weeks’ gestation, but also postpartum at 6 months after delivery. Additionally, the large sample size and fixed ultrasound device settings are the key elements of this study. The key element in reproducing our echogenicity measurements is the use of identical system settings. Most segmentation of the TUIs and analyses of the echogenicity is done by the computer, decreasing the observer variability.

When comparing our study results with those of the current literature we found that measuring echogenicity of the puborectal muscle is new in urogynaecology, but has been shown to be clinically relevant in other fields of medicine, for instance, as an indicator of rotator cuff partial-thickness tear or tendinopathy of the shoulder and discrimination between myopathies and neuropathies in children [12, 14]. In the current literature Weinstein and co-workers reported puborectalis muscle areas of 4.8 cm^2^ ± 2.4 at rest and 5.3 cm^2^ ± 2.1 during contraction [30]. In both manoeuvres our results are slightly higher. One explanation could be that we studied nulliparous pregnant women, whereas Weinstein and coworkers studied only non-pregnant women. Another explanation could be that they measured the PMA by subtracting the inner from the outer hiatal area, instead of using the numbers of pixels in the delineated muscle area for the calculation. In our previous paper we described that the mismatch between two measurements occurred along the border of the delineated area, and was between 8 and 15 %.

In conclusion, this study shows that puborectalis muscle echogenicity, as an indicator of muscle composition, significantly changes during pregnancy and after delivery. The next step is to investigate whether these changes might be associated with urogenital symptoms or pregnancy outcome. As measuring echogenicity has been shown to be of practical use in other fields of medicine, further exploring its potential value seems warranted. The most critical issue remains the use of identical settings of the software to be able to compare future research.

## References

[1] Jelovsek J, Maher C, Braber M (2007). Pelvic organ prolapse. Lancet.

[2] Slieker-ten Hove M, Pool-Goudzwaard A, Eijkemans M, Steegers-Theunissen R, Burger C, Vierhout M (2009). Symptomatic pelvic organ prolapse and possible risk factors in a general population. Am J Obstet Gynecol.

[3] Dietz H, Bernardo M, Kirby A, Shek K (2001). Minimal criteria for the diagnosis of avulsion of the puborectalis muscle by tomographic ultrasound. Int Urogynecol J.

[4] Dietz H (2010). Pelvic floor ultrasound: a review. Am J Obstet Gynecol.

[5] Dietz HP, Shek C, Clarke B (2005). Biometry of the pubovisceral muscle and levator hiatus by three-dimensional pelvic floor ultrasound. Ultrasound Obstet Gynecol.

[6] Dietz HP (2007). Quantification of major morphological abnormalities of the levator ani. Ultrasound Obstet Gynecol.

[7] Lanzarone V, Dietz HP (2007). Three-dimensional ultrasound imaging of the levator hiatus in late pregnancy and associations with delivery outcomes. Aust N Z J Obstet Gynaecol.

[8] Shek KL, Kruger J, Dietz HP (2012). The effect of pregnancy on hiatal dimensions and urethral mobility: an observational study. Int Urogynecol J.

[9] Van Delft K, Thakar R, Shobeiri SA, Sultan AH (2014). Levator hematoma at the attachment zone as an early marker for levator ani muscle avulsion. Ultrasound Obstet Gynecol.

[10] Grob ATM, Veen AAC, Schweitzer KJ, Withagen MIJ, van Veelen GA, van der Vaart CH (2014). Measuring echogenicity and area of the puborectalis muscle: method and reliability. Ultrasound Obstet Gynecol.

[11] Pillen S, Verrips A, van Alfen N, Arts IM, Sie LT, Zwarts MJ (2007). Quantitative skeletal muscle ultrasound: diagnostic value in childhood neuromuscular disease. Neuromuscul Disord.

[12] Maurits NH, Beenakker EAC, Van Schaik DEC, Fock JM, van der Hoeven JH (2004). Muscle ultrasound in children: normal values and application to neuromuscular disorders. Ultrasound Med Biol.

[13] Maurits NM, Bollen AE, Windhausen AEJ, de Jager A, van der Hoeven JH (2003). Muscle ultrasound analysis: normal values and differentiation between myopathies and neuropathies. Ultrasound Med Biol.

[14] Sofka CM, Haddad ZK, Adler RS (2004). Detection of muscle atrophy on routine sonography of the shoulder. J Ultrasound Med.

[15] Ramanah R, Berger MB, Parratte BM, DeLancey JO (2012). Anatomy and histology of apical support: a literature review concerning cardinal and uterosacral ligaments. Int Urogynecol J.

[16] Van Veelen GA, Schweitzer KJ, van der Vaart CH (2014). Ultrasound imaging of the pelvic floor: changes in anatomy during and after first pregnancy. Ultrasound Obstet Gynecol.

[17] Van Veelen A, Schweitzer K, van der Vaart H (2014). Ultrasound assessment of urethral support in women with stress urinary incontinence during and after first pregnancy. Obstet Gynecol.

[18] Unger CA, Weinstein MM, Pretorius DH (2011). Pelvic floor imaging. Am J Obstet Gynecol.

[19] Dietz HP, Shek C, de Leon J, Steensma AB (2008). Ballooning of the levator hiatus. Ultrasound Obstet Gynecol.

[20] Mayans D, Cartwright MS, Walker FO (2011). Neuromuscular ultrasonography: quantifying muscle and nerve measurements. Phys Med Rehabil Clin N Am.

[21] Herrera E (2000). Metabolic adaptations in pregnancy and their implications for the availability of substrates to the fetus. Eur J Clin Nutr.

[22] Shulman GI (2014). Ectopic fat in insulin resistance, dyslipidemia, and cardiometabolic disease. N Engl J Med.

[23] Reimers K, Reimers CD, Wagner S, Paetzke I, Pongratz DE (1993). Skeletal muscle sonography: a correlative study of echogenicity and morphology. J Ultrasound Med.

[24] Hofer M (2005). Ultrasound teaching manual: the basics of performing and interpreting ultrasound scans.

[25] Grob ATM, Veen AAC, Schweitzer KJ, Withagen MIJ, van der Vaart CH (2014) Assessment of puborectalis muscle echogenicity: effect of BMI, age, sport activity and ultrasound probe pressure. Oral presentation presented at the annual world congress meeting of the International Society of Ultrasound in Obstetrics and Gynecology, Barcelona, Spain

[26] Madaras EI, Perez J, Sobel BE, Mottley JG, Miller JG (1988). Anisotropy of the ultrasonic backscatter of myocardial tissue. II. Measurements in vivo. J Acoust Soc Am.

[27] Volløyhaug I, Mørkved S, Salvesen Ø, Salvesen KA (2015). Pelvic organ prolapse and incontinence 15–23 years after first delivery: a cross-sectional study. BJOG.

[28] Gyhagen M, Bullarbo M, Nielsen TF, Milsom I (2013). Prevalence and risk factors for pelvic organ prolapse 20 years after childbirth: a national cohort study in singleton primiparae after vaginal or caesarean delivery. BJOG.

[29] Orejuela FJ, Shek KL, Dietz HP (2012). The time factor in the assessment of prolapse and levator ballooning. Int Urogynecol J.

[30] Weinstein MM, Jung SA, Pretorius DH, Nager CW, den Boer DJ, Mittal RK (2007). The reliability of puborectalis muscle measurements with 3-dimensional ultrasound imaging. Am J Obstet Gynecol.

